# Farther Account of the Bark Described in the Preceding Article

**Published:** 1789

**Authors:** Alexander Williams

**Affiliations:** Physician in Trinidad


V. *Farther Account of the Bark described in the preceding Article; being an Extract of a Letter from* Alexander Williams, *M. D. Physician at Trinidad. Communicated to Dr.* Simmons *by Mr.* William Blizard, *F. R. S. and S. A. Surgeon of the London Hospital.*

THE bark in queftion is brought to us by the Spaniards from Anguftura in South America, packed in ftraw, in pieces from one to two feet in length, and from an inch to an inch and a half in width.

It is of a brownifh yellow colour; has a raw unpleafant fmell, and a very difagreeable bitter tafte, without any aromatic warmth. Its raw fmell, however, I attribute entirely to its frefhnefs, as it lofes it in a great meafure, if not wholly, by being dried in the fun or baked over a gentle heat, and even acquires in its room fomewhat of an aromatic one, and the bitter becomes lefs difagreeable.

It gives out readily to either a watery or fpirituous menftruum, tinging the fluid of a pale gold colour. It is in one of thefe modes, chiefly however in the latter, that our planters ufe it among their negroes in fever, and pains of the belly, ftomach, &c.

The tree from which it is got is not yet known here; but we hope ſoon to become acquainted with it, as ſome of our botanical gentlemen have written for the flowers, &c., in order to inveſtigate it thoroughly.

The virtues of this bark ſeem to be pretty ſimilar to thoſe of the Peruvian bark; in ſome of theſe it has the advantage even over the latter, particularly in that of putting a ſtop to the paroxyſms of an intermittent fever more ſpeedily, leſs quantity of it being required, ſeldom more than ſix or eight doſes being neceſſary; nay, I am told by ſome gentlemen, that a ſingle doſe has often had the deſired effect.

It has, too, this farther advantage, that it does not cauſe that diſagreeable ſenſe of weight and fulneſs in the ſtomach, with coſtiveneſs, which the Peruvian bark moſt frequently occaſions, but keeps the belly gently open. It is found of the greateſt ſervice in diarrhœas, dyſenteries, and other complaints of the inteſtines, which the negroes are ſo ſubject to; and I believe will always prove uſeful in every diſorder ariſing from laxity and want of tone in the muſcular fibre. As an external application I have little or no experience of its effects. I ſhould be much ſurpriſed, however, if it ſhould not prove equally equally uſeful as it has done as an internal one. I am confident great advantage may be expect-ed from it when externally applied to gangrene, old flaccid ulcers, and the like complaints.

Dr. Ewer aſſures me he has ſeen the beſt ef-fects from an external application of it in a caſe of fever that was highly putrid. In this caſe the whole ſkin had already become diſcoloured with livid ſpots, a mortification had taken place in the throat, and even the black vomiting and hiccough had come on.

I will not at preſent ſay any thing more than merely what relates to my own caſe, and then leave you to judge whether this new remedy does not deſerve your and every other medical perſon's candid trial.

About a month ago, after much previous fa-tigue, and expoſure to noxious effluvia, I was ſeized with fever. I immediately took ſome emetic tartar, and diſcharged a great quantity of bile; after which the fever intermitted: being very coſtive, however, I thought it proper to take ſome aperient medicine; but before this had begun to operate my fever returned, at-tended with a violent pain in the ſide and great difficulty of breathing, for which I applied a bliſter to the affected part, and took ſome more emetic emetic tartar; the fever then again intermitted, and I immediately began the uſe of the Peruvian bark, not being yet acquainted with this new bark. I found, however, that the fever, notwithſtanding the uſe of the bark, aſſumed a tertian type, and continued ſo for three weeks, when I was adviſed to make trial of this new bark; which I did, and by its means got rid of the fever immediately, it having returned but once after its uſe. A few days ago, after ſitting up two or three nights ſucceſſively, I was again attacked with fever; but not having any of the new bark by me, I had recourſe to the Peruvian bark; of which, however, I could not take more than four doſes before I was obliged to diſcontinue its uſe from the diſagreeable ſenſe of weight and fulneſs that it cauſed: indeed I rejected the fifth doſe; upon which I ſent to Dr. Ewer, and procured a little of the new bark, and took a ſtrong infuſion of it with Madeira wine during one day, which put a final ſtop to the fever.

